# Organized Violence and Institutional Child Delivery: Micro-Level Evidence From Sub-Saharan Africa, 1989–2014

**DOI:** 10.1007/s13524-018-0685-4

**Published:** 2018-06-13

**Authors:** Gudrun Østby, Henrik Urdal, Andreas Forø Tollefsen, Andreas Kotsadam, Ragnhild Belbo, Christin Ormhaug

**Affiliations:** 1 Peace Research Institute Oslo (PRIO), PO Box 9229, Grønland, NO-0134 Oslo, Norway; 2 The Ragnar Frisch Centre for Economic Research, University of Oslo, Gaustadalléen 21, 0349 Oslo, Norway; 3 Noragric, International Environment and Development Studies, Norwegian University of Life Sciences, PO Box 5003, NO-1432 Ås, Norway

**Keywords:** Armed conflict, Demographic and Health Surveys, Maternal health, Organized violence, Sub-Saharan Africa

## Abstract

The conditions under which a mother gives birth greatly affect the health risk of both the mother and the child. This article addresses how local exposure to organized violence affects whether women give birth in a health facility. We combine geocoded data on violent events from the Uppsala Conflict Data Program with georeferenced survey data on the use of maternal health care services from the Demographic and Health Surveys. Our sample covers 569,201 births by 390,574 mothers in 31 countries in sub-Saharan Africa. We use a mother fixed-effects analysis to estimate the effect of recent organized violence events within a radius of 50 km of the home of each mother on the likelihood that her child is born in a health facility. The results indicate that geographical and temporal proximity to organized violence significantly reduces the likelihood of institutional births. Although the level of maternal health care overall is lower in rural areas, the negative effect of violence appears to be stronger in urban areas. The study further underscores the importance of household and individual resilience, indicating that the effect of organized violence on institutional child delivery is greater among poor and less-educated mothers.

**Electronic supplementary material:**

The online version of this article (10.1007/s13524-018-0685-4) contains supplementary material, which is available to authorized users.

## Introduction

One of the targets of the Sustainable Development Goals is to reduce the global maternal mortality rate to less than 70 per 100,000 live births by 2030. The most recent years have seen some improvements in maternal health, but progress is still slow, particularly in sub-Saharan Africa (SSA). A recent review put the odds that a woman in SSA will die from complications related to pregnancy and childbirth at 1 in 20 compared with 1 in 6,250 in the developed world (United Nations 2012). In a region where the majority of countries have experienced armed conflict since the end of the Cold War (Melander et al. 2016), we argue in this article that this poor performance in terms of maternal survival in part be due to detrimental effects of armed conflicts on maternal health.

The key to improved maternal and reproductive health (MRH) is the provision of adequate services, such as professional birth assistance (Van Rijsbergen and D’Exelle 2013). We argue that conflict may negatively affect institutional child delivery. Studies have indeed identified limited access to health care as one important explanation for excess female mortality also in war-torn countries (Ormhaug 2014; Urdal and Chi 2013), but no study has yet assessed the effect of conflict on institutional child delivery at the local level on a larger cross-national scale, with a suitable design making causal inference possible. With this article, we aim to fill that gap.

A key weakness of existing cross-national comparisons is that armed conflicts rarely affect entire countries equally but are typically confined to limited geographical areas (Buhaug and Rød 2006). Moreover, as we show in this article, institutional child delivery tends to be highly uneven within countries (see also Countdown Group 2008). Hence, studies of country-level maternal health aggregates may lead to an ecological fallacy by incorrectly deducing inferences from these aggregate studies about individual health care–seeking behavior during conflict. We overcome this problem by using geographical information systems (GIS) to combine detailed georeferenced individual-level data on institutional child delivery from Demographic and Health Surveys (DHS) and subnational data on conflict events and fatalities from Uppsala Conflict Data Program (UCDP)/Peace Research Institute Oslo (PRIO) (Sundberg and Melander 2013).

A growing body of micro-level studies has focused on various demographic consequences and responses to armed conflict (Agadjanian and Prata 2002; Bohra-Mishra and Massey 2011; Lindstrom and Berhanu 1999; Verwimp et al. 2009; Williams et al. 2012). However, to the best of our knowledge, our study represents the first systematic micro-level investigation of how local conflict patterns affect the use of maternal health care services across several countries.

Our findings indicate that women who live in locations that are geographically and temporally proximate to more intense organized violence have a significantly lower probability of giving birth in a health facility. A rough estimation suggests that organized violence in SSA causes approximately 47,000 children to be born outside health facilities every year. The importance of contextual factors indicates the prospects for resilience. Although the level of institutional child delivery is generally lower in rural areas, the negative effect of conflict seems to be stronger in urban areas. Also, the negative effect of violent events on institutional delivery is stronger for households with low levels of wealth and for mothers with low levels of education.

## Conflict, Health, and Gender: Literature Review

Ongoing debate within the research community concerns overall health effects of war, or *excess mortality*. This term captures negative health effects that extend beyond deaths attributable to battle—including mortality stemming from the overall deterioration of the social, economic, and political fabric—translating into total war deaths that would not have happened in the absence of war. Estimating the overall health effects in conflict areas is fraught with uncertainty, controversy, and methodological challenges, and collecting relevant data is typically not a priority during the chaos of a conflict (Murray et al. 2002).

Ghobarah et al. (2003) claimed that the indirect effects of civil conflict are underestimated not least due to the neglect of long-term effects. Plümper and Neumayer (2006) analyzed gender-specific cross-national life expectancy data and found that the most severe armed conflicts tended to decrease the gap between female and male life expectancy, supporting their hypothesis that severe armed conflicts, on average, affect women more adversely than men. Although the authors mentioned maternal mortality as one likely causal mechanism (Plümper and Neumayer 2006:38), the proposition was not tested explicitly. Li and Wen (2005) found that the immediate effects of conflict on adult mortality rates were higher for men than for women, whereas the long-term consequences were significant and equally important for women and men. Finally, in a study of African mortality, Iqbal (2010) found that women were slightly more affected than men by low-intensity conflict. All these country-level studies have stopped short of empirically investigating the actual reasons for excess female mortality.

Overall, studies assessing cultural and political determinants of maternal mortality are lacking (Gil-González et al. 2006). One notable exception is a study of 42 African countries by O’Hare and Southall (2007), which reported that maternal mortality ratios (MMRs) were 45 % higher in post-conflict countries than in nonconflict countries. However, they did not control for other factors that are generally associated with higher risk of conflict. In a global study of developing countries, Urdal and Chi (2013) found that armed conflicts are associated with higher overall MMRs, but they did not assess the effects of conflict on the use of MRH services.

In addition to cross-national studies, several case studies have evaluated how reproductive health care is affected by conflict. Kottegoda et al. (2008) reported higher levels of poverty, early marriage, and higher maternal mortality among conflict-affected women in Sri Lanka. High MMRs were also reported in parts of Burma, where the military junta had attempted to cut off all resources, and alternative ways of delivering health care had to be sought (Mullany et al. 2008). Chandrasekhar et al. (2011) showed that the conflict in Rwanda led to a decrease in the number of births given in a health facility. Some populations affected by conflict may, on the other hand, experience an improvement in health. For example, refugees and internally displaced persons (IDPs) living in camps that receive the attention of international or local health providers have been found to be as well or even better off than both people in their home communities and noncamp neighboring populations (Howard et al. 2008)

In sum, little research has investigated the effects of organized violence on maternal health, and findings of those rare studies do not point in one clear direction. Part of the problem may be the lack of systematic subnational studies that address the local dynamics of the conflict–maternal health relationship across countries. Geographically disaggregated studies and micro-level evidence are crucial because both conflict patterns and access to services vary significantly within countries. To our knowledge, the current study is the first to disaggregate patterns of organized violence and the use of maternal health care services across several countries.

## Organized Violence and Institutional Child Delivery

During armed conflict, women face myriad challenges that may affect their use of MRH services (Al Gasseer et al. 2004:9; Black et al. 2014). Local exposure to organized violence can negatively affect access to reproductive and maternal health services both directly and indirectly: directly, through severing the provision of such services; and indirectly, through making it more difficult to reach health facilities, forcing people away from their homes, and forcing the long-term breakdown of social institutions, which makes it harder for certain groups to make use of the health system.

The mechanisms through which violent conflict affects the use of maternal health care can broadly be classified as either demand or supply effects (Price and Bohara 2013). At the most basic level, violent conflict may increase the risk associated with travel. As it becomes less safe to travel to health facilities, institutional child delivery is likely to decrease. Further, conflict may negatively affect household income and hence potentially reduce the demand for health care services, which could involve user fees and thus be costly. Adding this to increased travel time and personal risk, and possibly increased waiting time at the medical facility if there is a decrease in medical personnel, the opportunity costs associated with going to the hospital is likely to increase. On the supply side, the provision of maternal health care may be reduced directly if facilities are destroyed or the medical personnel has fled, are killed, or are imprisoned. A reduction in supply of personnel and medical equipment is further likely to lead to increased fees for MRH services, such as birth assistance.

We can think of three theoretical mechanisms through which conflict is likely to have a negative effect on institutional child delivery: (1) disturbing and changing population movement patterns, including causing refugee movements; (2) severely undermining economies both locally and nationally; and (3) destroying infrastructure, including health centers, hospitals, and roads. In principle, each of these mechanisms may be tested empirically. However, detailed local and comparable information on population movement, economic development, and access to health infrastructure is unavailable for the spatiotemporal domain covered by the current analysis. Furthermore, such data are also particularly challenging to collect during periods of violent conflict.

Hence, we leave for future, more-detailed case studies to further test and corroborate the various proposed mechanisms. For the current analysis, we instead propose the following general hypothesis:

- *Hypothesis 1:* The more exposed a mother is to organized violence in her home area, the lower the likelihood that she will give birth in a health facility.

Even in conflict-ridden areas, several socioeconomic factors are assumed to determine the resilience of women with respect to health care–seeking behavior (McGinn 2000). We expect that these factors affect individuals’ and communities’ capacity to adapt to increasingly complex social, political, and economic environments. Key socioeconomic factors—such as rural/urban residence, wealth, and education—have been found to greatly affect the use of maternal health care services.

Women in urban areas are generally far more likely to receive maternal health care than women in rural areas (Fapohunda and Orobaton 2014; Magadi et al. 2007; Pathak et al. 2010). We expect that urban women will be less affected by armed conflict than rural women given that the supply of health care in urban areas is higher, which should mean that urban women more easily could shift from one supplier to another in the case of service disruption. Women in rural areas, who typically rely on one or few suppliers, are likely more vulnerable to armed conflict:

- *Hypothesis 2*: The negative effect of organized violence on the likelihood of giving birth in a health facility is stronger in rural than in urban areas.

Another well-established finding is that wealthier women receive better care (Fapohunda and Orobaton 2014; Pathak et al. 2010; UNICEF 2008). We expect that women from wealthier households, who are able to purchase access to private health care, will be less vulnerable to service disruption due to armed conflict:

- *Hypothesis 3:* The negative effect of organized violence on the likelihood of giving birth in a health facility is stronger for mothers from poorer households than for mothers from more affluent households.

Furthermore, educated mothers usually have healthier families overall, and previous studies using DHS data have shown that educated mothers are more likely than uneducated mothers to have access to professional delivery care (Bell et al. 2003; Fapohunda and Orobaton 2014; Pathak et al. 2010). We assume that education improves a woman’s ability to access information about service provision in unstable situations and that increasing education thus should translate into lower susceptibility to armed conflict:

- *Hypothesis 4:* The negative effect of organized violence on the likelihood of giving birth in a health facility is stronger for less-educated mothers than for more-educated mothers.

## Empirical Strategy

To explore whether and how organized violence affects institutional child delivery, we link survey data from Demographic Health Surveys (DHS)^1^ with violent events data from the Uppsala Conflict Data Program (UCDP) Georeferenced Event Dataset (GED) (Sundberg and Melander 2013). The DHS surveys include information on respondents’ fertility behavior and use of various maternal health services covering all births within five years prior to the survey.

In each DHS, a sample of households is selected throughout the entire country. Women aged 15–49 are interviewed about health, nutrition, family, and other demographic factors. The survey instrument also includes a number of additional items, such as ethnicity, education, and household assets. Typically, DHSs cover approximately 7,000–10,000 respondents nationally, representing urban and rural areas and provinces/states. DHS surveys are conducted every four to five years in most countries, with the same questions asked in each survey to facilitate comparisons across time and space.

Several of the DHS surveys include detailed information about the specific location of each sample cluster (village/town/city). Because we are interested in the location of each respondent and her spatial relationship with conflict,^2^ we use only the DHSs containing GPS coordinates.^3^ We use the coordinates of the respondent’s cluster to identify whether conflict has taken place in the respondent’s proximity.

**Fig. 1:**
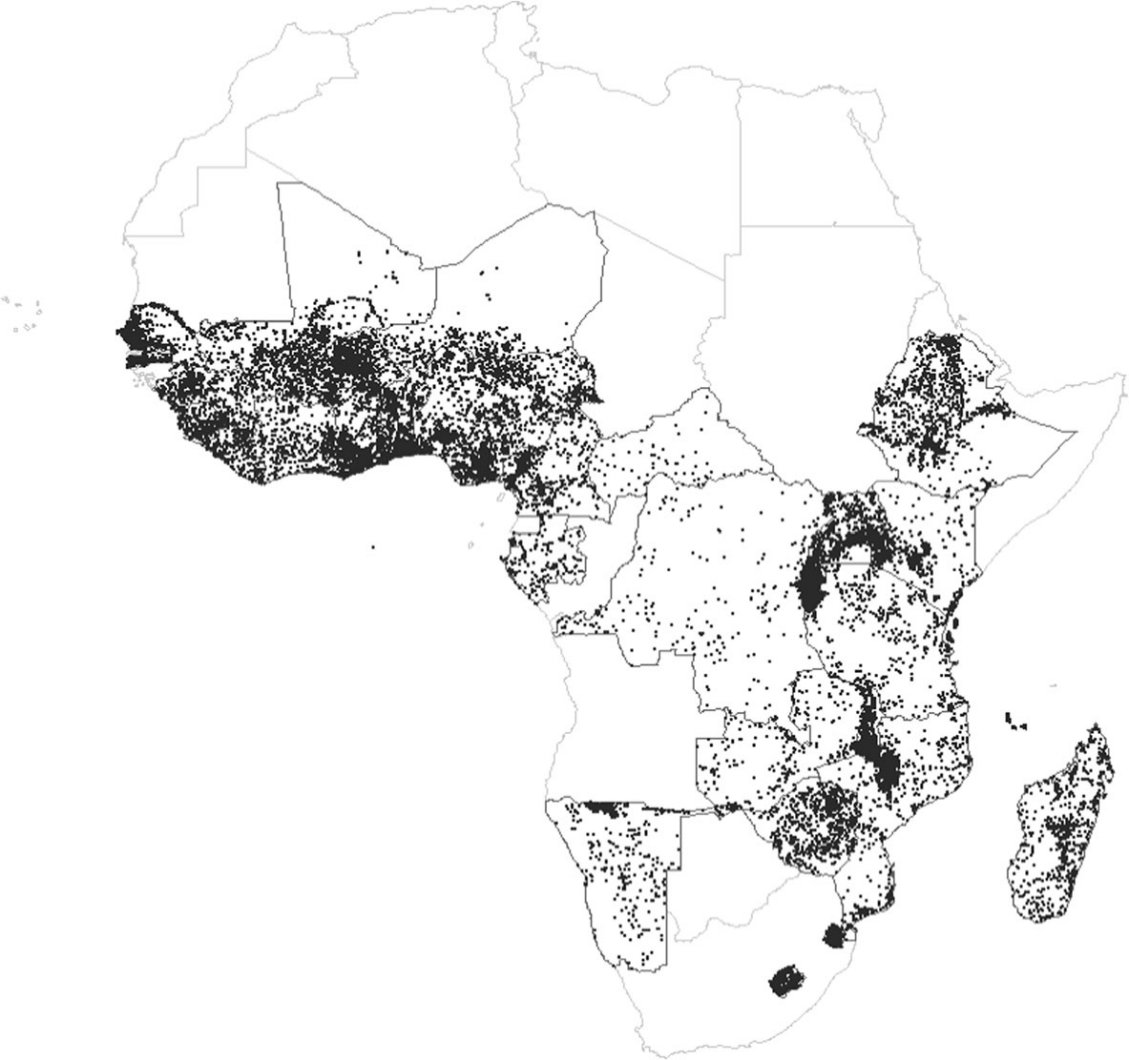
Location of DHS survey sites in sub-Saharan Africa, 1990–2014. Black dots represent DHS cluster locations

In total, our full merged data set includes 72 surveys in 31 SSA countries^4^ covering 28,325 cluster locations and 747,746 individual respondents, of which 394,255 mothers gave birth to a total of 569,201 live children in the period 1989–2014 (during the five-year period preceding each survey).^5^ Fig. 1 shows the spatial distribution of the DHS clusters, or sites, for all surveys combined.

### Dependent Variable: Institutional Delivery

One of the most common measures of access to maternal health care services is the use of institutional deliveries. Our dependent variable, *institutional child delivery*, takes the value 1 if the delivery happened in a medical health facility (including all types of public and private hospitals, clinics, health centers, health posts) and 0 otherwise.^6^

In this study, we are primarily interested in the local effects of organized violence on institutional child delivery, but this seems crucial for maternal death.^7^ To test its validity, we regress our key variable of interest—institutional child delivery—on MMR in Table 1,^8^ and find that it is negatively associated with maternal deaths at the national level. We believe it is safe to assume that the same is true at the subnational level.

**Table 1:** The effect of institutional child delivery on maternal mortality rate (MMR): 31 African countries, 1987–2014

	MMR
Variables	(1)
Institutional Child Delivery	–413.320**
	(134.063)
Number of Observations	398
*R* ^2^	.775
Country Fixed Effects	Yes
Year Fixed Effects	Yes
Mean in Sample	615.6

*Notes:* Results are linear regressions. Robust standard errors are shown in parentheses.

**p < .01

**Fig. 2:**
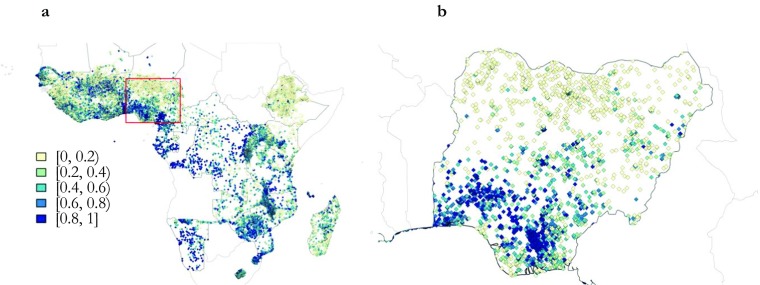
Delivery in a medical facility in SSA, share (%) by survey cluster, various years. The sources of panel a are all DHS surveys listed in Table 4 in the appendix, and panel b zooms in on Nigeria

The maps in Fig. 2 show the geographical distribution of delivery in a health facility by survey cluster for the last born in the year preceding each survey. In the SSA map (Fig. 2, panel a), clear patterns are difficult to discern at first glance, although institutional births seem to be more common in the western and southern parts of SSA. However, we have an unbalanced panel with survey points from the years 1989–2014. Hence, looking at the distribution within a single country may prove more interesting. The map in panel b shows the distribution of deliveries in a medical facility by survey cluster point in Nigeria. The darker the color, the higher the percentage of women reporting that their last-born was delivered at a medical facility. The pattern is clear: women in northern regions seem to have much lower access to institutional delivery than women in southern Nigeria.^9^

### Independent Variable: Organized Violence

To identify whether a DHS respondent has experienced organized violence in her vicinity prior to a given pregnancy/delivery, we rely on the UCDP GED (Croicu and Sundberg 2016; Sundberg and Melander 2013).^10^ The UCDP GED includes information on the location of organized violence events and the number of deaths caused by each event. Events are included for all UCDP conflict dyads^11^ that crossed the 25-deaths threshold in any year of the UCDP annual data. Hence, the UCDP GED defines an event as “an incident where armed force was by an organised actor against another organized actor, or against civilians, resulting in at least 1 direct death at a specific location and a specific date” (Croicu and Sundberg 2016:2).^12^

The UCDP GED data include information on three types of organized violence: (1) state-based conflict (between two states or one state and one or more rebel groups); (2) nonstate conflict (between two organized actors, neither of which is the government of a state); and (3) one-sided *violence* (by an organized armed group against civilians). Although we expect conflict events across all three types to have largely similar effects on institutional births, we unpack the independent variable and look at separate effects on institutional births for state-based, nonstate, and one-sided violence in Online Resource 1 (see Table S17).

**Fig. 3:**
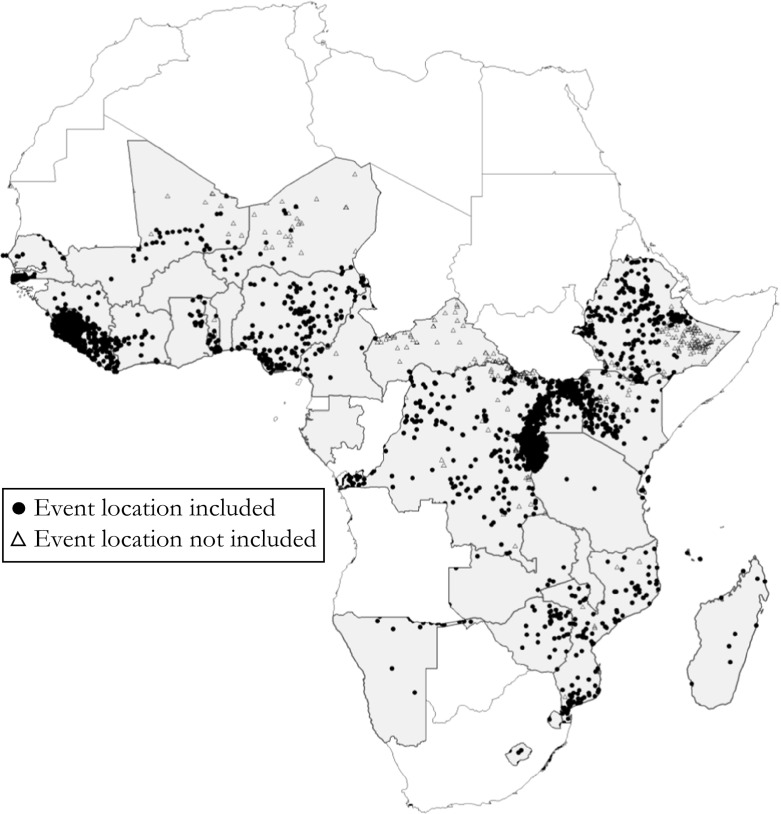
Included and nonincluded events of organized violence in sample countries, 1989–2014. Fig. S3 in Online Resource 1 shows a similar map including DHS buffer zones. Fig. S4 graphs the different event types over time. Figs. S5–S6 show maps disaggregated by type of organized violence

Fig. 3 maps all organized violence events in the UCDP GED data for 1989–2014 for the countries included in the following analysis. Point coordinates represent each event, and the location has been retrieved using news reports and georeferenced using global gazetteers. The map distinguishes between events included in creating our conflict event measure and events not included because they fall outside the 50 km inclusion criteria. Because multiple events may overlap at the same location, we place included events on top.

Our main independent variable—violence intensity—is an event count for all types of violence combined within a given spatiotemporal domain (as described shortly). As an alternative measure of violence intensity, we also use a count of the number of conflict-related deaths. The event count is our preferred measure because we believe that repeated violent events in a local setting may have stronger security implications on the ground than fewer, more-deadly events.

The UCDP GED data capture the individual events associated with large-scale organized violence, such as the state-based conflicts in the Democratic Republic of the Congo (DRC) and Ethiopia-Eritrea, as well as one-sided violence like the Rwandan genocide. Only a small handful of SSA countries have escaped organized violence entirely in the period since 1989. Among the less well-known cases is Kenya, in which more than 5,000 people have been killed in organized violence between 1989 and 2016, more than 95 % in nonstate and one-sided violence. Another is Senegal, where more than 2,000 people have been killed across all three types of violence, although the majority were killed in state-based conflict. Although the Senegalese organized violence was most intense in the 1990s, violent events have been recorded almost every year for the last 15 years with available data (i.e., 2001–2016). Zimbabwe has experienced recent low-intensity, yet widespread, one-sided violence related both to electoral campaigns and to government control over alluvial diamond fields. Across the period and space covered by the current analysis, nonstate conflict events make up 17 % of all events in SSA, one-sided events account for 31 %, and state-based events account for 51 %.

Assessing the effect of organized violence on institutional deliveries requires a unit of observation capable of intersecting the location of the DHS locations with the locations of subnational violence events. We employ GIS^13^ to expand the DHS points to polygons using spatial buffering at given radii (de Smith et al. 2007), permitting the identification of the shared spatial relationship between the DHS cluster location and the event locations. For the main analysis, this buffer zone is set to a radius of 50 km, which is arguably a reasonable distance for which violent events could be expected to affect the availability of local maternal health care services. However, because there is no theoretical or empirical prior guiding this choice, we also try 25 km and 100 km radii as robustness checks. We are then able to identify the number of events intersecting with the respondents’ buffer zone.

**Fig. 4:**
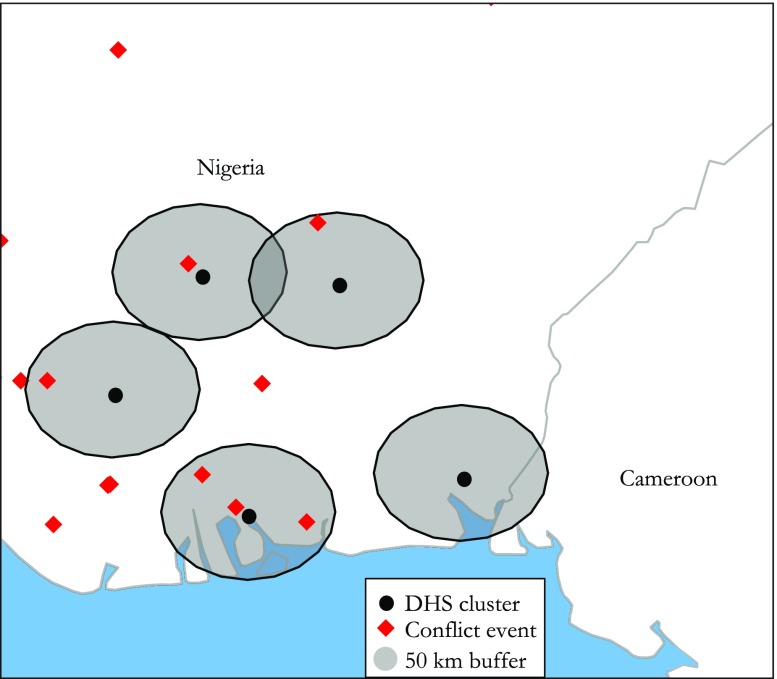
Buffer (50 km) around selected DHS clusters in Southwest Nigeria (2008), with violent events six months prior to the survey

Figure 4 illustrates the data design, where survey locations are assigned a 50 km buffer. Events are counted if they intersect with the buffer only. To identify the spatial and temporal effect of organized violence with a range of health outcomes for each birth, we count the number of organized violence events within the 50 km buffer zones in the six-month period prior to the individual birth date. This time frame has two advantages. First, it implies fewer problems with migration patterns: the longer the time span, the more likely that the mother has lived elsewhere. Second, the six-month temporal window eliminates the possibility of selection into childbearing.^14^

The number of violence events within the 50 km buffer six months prior to each birth date ranges from 0 to 129. However, the distribution is highly skewed: 94.04 % of the sample did not experience a recent conflict event within a 50 km radius. To account for this, we use a log-transformed variable of violent events in the main statistical models to follow. In the alternative models, we use the log-transformed number of conflict-related deaths.

### Other Moderating Independent Variables

We construct several variables from the DHS that we argue might influence the effect of conflict on institutional child delivery: an urban indicator (urban/rural), mother’s education level, and household wealth.

The urban indicator is dichotomous and indicates whether a household is situated in a rural or urban area as defined by DHS. Education is measured as the mother’s completed education years. Because the DHS do not include data on income, we construct an additive household asset index, combining information on the ownership of various household assets (electricity, refrigerator, radio, television, bicycle, motorcycle, and car), following Østby (2008). The asset index ranges from 0 (respondent household owns none of the listed household assets) to 1 (respondent household possesses all the mentioned assets). Descriptive statistics for all dependent and independent variables, including breakdowns by rural and urban areas, are provided in Tables 5 and 6 in the appendix.

### Statistical Model

Given that fertility levels in Africa are high and organized violence is relatively common, we observe a sizable number of women giving birth both before and after conflict. This implies that we are able to run regressions with mother fixed effects. Thus, we estimate effects of conflict using only within-mother variation—meaning, mothers who have given birth to at least two children in the previous five years—where there is variation in the conflict events variable across the births. The advantage of such a design—over, for example, cross-country or even within-country regression analyses—is that we are able to control for a vast number of variables that may otherwise be spuriously correlated with both maternal care and conflict. In fact, our approach implies controlling for all the observed and unobserved factors that are fixed over time for each woman. It also ensures that the estimated effect is not driven by endogenous population changes. This worry looms large in empirical analyses of both conflict and health. The disadvantage of using the method is that it requires a vast sample and that the results are not generalizable to mothers that give their first birth after the conflict.

To investigate the effects of organized violence on institutional delivery, we estimate the following linear probability model:

1 $$ {Y}_{imcb}={\upalpha}_m+{Conflict}_{c\left(b-x\right)}+{\uplambda}_t+{\boldsymbol{\uptheta}}_{it}+{\upvarepsilon}_{imcb}, $$

where *Y* is the outcome for child *i* born by mother *m* in cluster *c* in birth month *b*. The mother fixed effects, α_*m*_, ensure that we are comparing the effects of sibling births with conditions that are as similar as possible except for the conflict that occurs in period *b* – *x*, where *x* is six months in the main specification. By including year-of-birth fixed effects, λ_*t*_, we are essentially estimating a difference-in-difference model wherein we first take the difference over time for the same mother before and after conflict, and then we take the difference in that difference between mothers giving birth in the same year but who are affected or not by a conflict. Additionally, we control for the time-varying variables in all regressions by adding the vector **θ**_*it*_. These variables, which by default vary by sibling, are birth order and a dummy variable for being part of a multiple birth sequence. We also control for the number of children a woman had in the previous five years. The standard errors are clustered at the level of the primary sampling unit so that we take into account that the observations are not independent within each cluster.

## Results

Is the likelihood of institutional delivery affected by the recent and proximate exposure to organized violence in a woman’s home area? Model 1 in Table 2 shows the baseline results for the main independent variable (logged number of conflict events during the last six months in a 50 km radius).^15^ The table shows a statistically significant negative relationship between organized violence and institutional births. Although assessing the overall toll of conflict on the use of maternal health services is challenging given the lack of detailed local data, our rough back-of-the-envelope estimation suggests that organized violence in SSA causes approximately 47,000 children to be born outside health facilities every year.^16^ This is admittedly a modest number relative to the total number of births on the continent, possibly giving credence to critical voices suggesting that the focus on maternal deaths in humanitarian settings is grossly overstated relative to the importance of extreme poverty (Nordenstedt and Rosling 2016:1865). However, two qualifications need to be made. First, this should be considered a lower-bound estimate because we are not capturing any long-term effects beyond 12 months after conflict or effects that extend beyond the geographical domain, and because some conflict-ridden countries (such as Somalia and Angola) are missing from the analysis. Because conflicts affect the access to maternal health care, the model further likely underestimates the number of births outside medical facilities because women in conflict areas have a higher risk of not surviving until the time of the survey. Second, the estimate captures the effects of conflict net of any interventions to ameliorate these consequences. The provision of health care in humanitarian settings—in particular, concentrated efforts in refugee camps—may even improve the health situation for those receiving assistance and for surrounding populations relative to the prewar situation.

**Table 2:** Organized violence and institutional birth: Baseline results

	(1)
Violent Events: Last Six Months (50 km radius)^a^	–0.009***
	(0.003)
Number of Observations	569,031
*R* ^2^	.008
Number of Mothers	390,484
Mother Fixed Effects	Yes
Year Fixed Effects	Yes
Mean in Sample	0.501

*Notes:* Results are linear regressions. Robust standard errors, clustered on DHS primary sampling unit, are shown in parentheses.

^a^Logged (controls for birth order and multiple births are not shown).

***p < .001

### Robustness of Baseline Results

We report several robustness checks in Tables S1–S17 in Online Resource 1. First, our results are robust with respect to the timing of the conflict events prior to the birth. In fact, the results are robust to both a larger (nine-month) and a smaller (three-month) window; they are also robust to using an alternative independent variable of birth assistance by medical professional rather than institutional birth, as shown in Table S1. Second, the results are robust to conflict exposure within different radii (25 km and 100 km), as shown in Tables S2 and S3. Third, as an alternative to using log-transformed number of conflict events, we test the effect of conflict when estimated in levels and estimated using a dummy variable for whether at least one conflict event occurred nearby. These results also look similar (see Tables S4 and S5).

We further attempt an alternative operationalization of violence intensity, using the log-transformed number of conflict-related deaths associated with previous events of organized violence rather than the event count itself (Table S6). Again, results remain similar.

Armed conflicts often spur migration. Another robustness check is to estimate the effects for a sample of women that we know lived in the same area (see Table S7). Unfortunately, this question is not asked in all surveys. Furthermore, the migration variables are problematic because we do not know the locations from which people moved. Given that people often move short distances, they were likely still affected by the conflict. Nonetheless, the results point in the same direction, and we cannot reject that they are in fact the same. Similarly, we can restrict the sample to women known to have lived in the area for at least five years. In sum, the results do not seem to be much affected by migration.

Further investigation of the issue of migration is presented in Table S8. First, we restrict the sample to the observations having information about migration but without using this information in any other way. The effect of conflict is not statistically significant in this sample, but the point estimates are similar. Thus, it is not migration per se that removes the statistical significance of the results but rather the decreased sample size. In column 2 in Table S8, we include an indicator variable for having missing information on migration. The difference between those having information on migration and those lacking such information is not statistically significant. Columns 3 and 4 further add interactions between different measures of migration and conflict, and there appears to be no statistically significant difference in the effect for migrants and nonmigrants. This also speaks to the mechanisms of the effects because we cannot conclude that the effect is driven by migration.

In our main specification, we cluster the standard errors at the DHS cluster level (i.e., at the level of the primary sampling unit), which is reasonable because we do not think that the observations are independent within each cluster. In fact, we know that everyone in the same cluster was affected by the same violent event. We can also cluster the standard errors at the country level and thereby allow for dependence also within a country, and Table S9 shows that the results are robust to such a clustering.

Finally, as shown in Table S10, when we exclude the countries that have no recorded instances of organized violence within 50 km and six months prior to any birth, the results remain largely unchanged.

### Heterogeneous Effects

As discussed in the theoretical section, we have reasons to believe that the effect of conflict differs across different areas and groups of the population. In Table 3, we present results for the tests of Hypotheses 2–4—that is, whether the effect of organized violence on the use of maternal health care services is conditioned by rurality, household welfare, or education.

**Table 3:** Organized violence and institutional births, heterogeneous effects

			Standardized Coefficients			
	Urban	Rural	Urban	Rural	Total Assets	Total Education
	(1)	(2)	(3)	(4)	(5)	(6)
Violent Events^a^	–0.012**	–0.008**	–0.011**	–0.006**	–0.013***	–0.013***
	(0.004)	(0.003)	(0.006)	(0.008)	(0.003)	(0.003)
Household Asset Index ($) × All Violence Events^a^					0.024*	
					(0.012)	
Education × All Violence Events^a^						0.001^†^
						(0.001)
Number of Observations	150,249	418,782	150,249	418,782	558,179	568,730
*R* ^2^	.004	.010	.004	.010	.003	.003
Number of Mothers	109,714	280,770	109,714	280,770	382,726	390,277
Mother Fixed Effects	Yes	Yes	Yes	Yes	Yes	Yes
Year Fixed Effects	Yes	Yes	Yes	Yes	Yes	Yes
Mean in Sample	0.770	0.405	0.770	0.405	0.499	0.501

*Notes:* Results are linear regressions. Robust standard errors, clustered on DHS primary sampling unit in all specifications, are shown in parentheses except in columns 3 and 4. Values in parentheses are *p* values for the standardized coefficients.

^a^Logged number of conflict events within 50 km radius six months prior to birth. Controls for birth order and multiple births are not shown.

^†^*p* < .10; *p < .05; **p < .01; ***p < .001

First, we split the sample by rural and urban clusters in Models 1 and 2. The effect of conflict on maternal health care is negative in both types of areas. At first glance, the effect seems to be larger in the urban areas, but because the means are higher in those areas, this is not necessarily the case. Hence, we also try standardized coefficients in Models 3 and 4 (i.e., where 1 standard deviation in X corresponds to Z standard deviation changes in Y). The effect still seems stronger in urban areas, which is contrary to our second hypothesis.

Second, we investigate the difference in the effects for poor and rich mothers by interacting the asset index with conflict exposure. Model 5 shows that the conflict has a larger negative correlation for the poorest mothers with no assets but no negative correlation at all for the richest individuals (because the statistically significant interaction term more than cancels out the negative baseline correlation). This is in line with our third hypothesis.

Finally, we explore whether the negative effect of armed conflict events on maternal healthcare is moderated by the mother’s education level. Model 6 reports the same type of analysis as earlier but with years of education instead of household assets. The advantage of this specification is that education is more likely to be predetermined. The results show that those with zero years of education are greatly affected, but those with 13 years of education are not at all affected by conflict. The results support our fourth hypothesis that the negative effect of armed conflict events on institutional deliveries is stronger for less-educated women.

In Online Resource 1, we further investigate the heterogeneity of our results. First, we study whether the main effect is stable across different periods (Table S11). In short, we find that the negative effects of organized violence are largest in the latter period, after 2004. Further, we test for heterogeneous effects across different levels of maternal health care service provision (Table S12) and find that our main result is driven by areas with a high share of births delivered in health facilities. Hence, despite an impressive expansion in maternal health care services around the world, our analysis shows that organized violence may lead to serious setbacks in the share of women giving birth at a health facility.

We also investigate the temporal dimension of the effects in a regression discontinuity. The results suggest a sudden drop in institutional deliveries precisely in the month when a conflict event happens followed by a rebound period of approximately three years before reaching pre-conflict levels (see Tables S14–S16).

## Discussion and Conclusion

Improvements in maternal health have been slower to materialize than foreseen when the targets for the Millennium Development Goals were set, and SSA is the region lagging the most behind. Whereas previous cross-national studies exploring maternal health in the region have employed a country-level framework and analysis (e.g., O’Hare and Southall 2007; Urdal and Chi 2013), this study uses geographically coded data to assess the relationship between armed conflict intensity and maternal care at the local/individual level. Pooling data on women from 72 DHS covering 31 countries in SSA and spatially linking these to detailed data on the timing and location of organized violence from UCDP GED (Sundberg and Melander 2013) allows us to study whether recent events in the immediate neighborhood of a woman affect her likelihood of giving birth at a medical facility.

Our main finding—that geographical and temporal proximity to organized violence significantly reduces the likelihood of institutional births—is not completely unexpected. However, improved maternal health care as a result of conflict and organized violence might be expected in some settings. For example, particularly violent locations may also attract more humanitarian aid, which may in turn improve the maternal health care relative to the pre-conflict situation. We again refer to the study by Howard et al. (2008), who indeed found that refugees and IDPs living in camps that receive the attention of international or local health providers fared as well as or even better than both people in their home communities and noncamp neighboring populations. Hence, ex-ante, it is not obvious that the total effect is negative; more importantly, existing research has not provided robust evidence in any direction.

Planning for effective interventions to improve maternal health in conflict and post-conflict settings requires providing sound empirical evidence concerning the relationship between organized violence and access to maternal health care services. The study presented herein represents an important step in this regard, advancing the field and the existent knowledge base in two main ways.

First, we provide the first systematic micro-level investigation of how local conflict patterns affect the use of maternal health care services across several countries, which enables generalizing the relationship between local exposure to organized violence and access to maternal health services.

Second, we have high confidence in our findings given our identification strategy. Using a quasi-experimental approach (mother fixed-effects analysis) allows us to control for a vast number of variables that may otherwise be spuriously correlated with both maternal care and organized violence. Apart from a randomized controlled trial, which is not feasible for ethical reasons, a mother fixed-effects study controlling for sibling-varying confounders is among the strongest types of causal inference strategies available to answer the question of whether recent and proximate armed conflict events may affect the likelihood that a child is born at a medical hospital. A likely reason why such quasi-experimental studies are seldom conducted is that they require very large sample sizes to obtain sufficient power to detect significant effects (Anekwe et al. 2015).

Our sample, consisting of as many as 569,201 births by 390,574 mothers, proves sufficiently large to detect a localized strong and statistically significant negative effect of exposure to organized violence on institutional births. This finding holds, subject to various robustness checks. A rough estimation indicates that this translates into approximately 47,000 fewer women giving birth at a medical facility in SSA every year because they live in a conflict-ridden area.

Although the level of maternal health care is generally lower in rural areas, the negative effect of organized violence seems to be stronger for women in urban areas, poor women, and women with less education. Finally, a sudden drop in institutional child delivery appears to occur precisely in the month a violent event happens. It takes approximately three years before institutional child delivery reaches pre-conflict levels.

Our findings present a call for further investigation. In particular, further studies should explore *why* organized violence reduces the likelihood of institutional births. As we alluded to earlier, the literature has proposed various mechanisms that may explain this relationship. First, civil violence will often lead to population movements. Initially, people are likely to gather in nearby locations that are perceived to be safe if the violence is expected to be of short duration, or they might move from their home location if conflict is intense and/or is expected to be long-lasting. Political violence and war are push factors for migration, and as an ultimate consequence of conflict, people will abandon their homes and end up as IDPs within their country or as refugees crossing into a neighboring state, often losing all their assets and becoming exposed to danger in the process. Earlier research has emphasized refugee movements as an important factor explaining the devastating effects of conflict (Bohra-Mishra and Massey 2011; Murray et al. 2002; Plümper and Neumayer 2006). If women are prevented from physically moving around their local community, or it becomes impossible to transport patients, this will impede institutional child delivery. Although minor violent events typically lead to short-term displacement—thus allowing people to return to their daily lives soon after the fighting is over—extended conflict activities may lead to long-term and substantial displacement, which is likely to severely affect institutional child delivery.

Second, intense armed conflicts are often highly disruptive to the economy (see World Bank 2011 for an overview). Economic decline at the national level is likely to decrease the financial capacity of states, channel resources away from health care, and make the use of funds for health care less efficient (Ghobarah et al. 2003). At the local level, conflicts may impede agriculture and interrupt trade, potentially leading to malnutrition and poverty. Because MRH services in developing countries are often fee-based, these factors combine to reduce the chances of receiving adequate care. These disruptions are not likely to subside immediately after a conflict is over but could linger for a long time.

Finally, destruction of infrastructures, such as health care facilities and roads, is a third factor emphasized in the literature (Li and Wen 2005; Murray et al. 2002). Health facilities may be targeted deliberately during conflicts in order to hurt an enemy population. Based on a systematic review of human rights reports in armed conflicts globally, Rubenstein and Bittle (2010:329) concluded that “assaults on patients and medical personnel, facilities, and transports, denial of access to medical services, and misuse of medical facilities and emblems have become a feature of armed conflict despite their prohibition by the laws of war.” Furthermore, in societies where health facilities are generally scarce and people are dependent on traveling extensively to receive professional care, conflicts may have a negative effect on health indirectly by damaging roads and means of transportation.

As explained earlier, with the data at hand in the current study, we are not able to test these various mechanisms. Further testing and corroborating these various proposed mechanisms is a task for future research. In particular, there is a need for detailed micro-level case studies. Moreover, future studies should explore the extent to which early external interventions to reach vulnerable populations during conflict may moderate the negative maternal health effect of the conflict.

### Electronic supplementary material

ESM 1 (DOCX 740 kb)

## Appendix

**Table 4:** Overview of DHS surveys used in analysis

Country	Survey Year	Country	Survey Year
Benin	1996	Madagascar	2008–2009
Benin	2001	Malawi	2000
Benin	2011–2012	Malawi	2004
Burkina Faso	1993	Malawi	2010
Burkina Faso	1998–1999	Mali	1995–1996
Burkina Faso	2003	Mali	2001
Burkina Faso	2010	Mali	2006
Burundi	2010	Mali	2012–2013
Cameroon	1991	Mozambique	2011
Cameroon	2004	Namibia	2000
Cameroon	2011	Namibia	2006–2007
Central African Republic	1994–1995	Niger	1992
Comoros	2012	Niger	1998
DRC	2007	Nigeria	1990
DRC	2013–2014	Nigeria	2003
Côte d’Ivoire	1994	Nigeria	2008
Côte d’Ivoire	1998–1999	Nigeria	2013
Côte d’Ivoire	2011–2012	Rwanda	2005
Ethiopia	2000	Rwanda	2010
Ethiopia	2005	Senegal	1992–1993
Ethiopia	2011	Senegal	1997
Gabon	2012	Senegal	2005
Ghana	1993	Senegal	2010–2011
Ghana	1998	Sierra Leone	2008
Ghana	2003	Sierra Leone	2013
Ghana	2008	Swaziland	2006–2007
Guinea	1999	Tanzania	1999
Guinea	2005	Tanzania	2010
Guinea	2012	Togo	1998
Kenya	2003	Uganda	2000–2001
Kenya	2008–2009	Uganda	2006
Lesotho	2004	Uganda	2011
Lesotho	2009	Zambia	2007
Liberia	2007	Zimbabwe	1999
Liberia	2013	Zimbabwe	2005–2006
Madagascar	1997	Zimbabwe	2010–2011

**Table 5:** Descriptive statistics

Variable	Number of Observations (births)	Mean	SD	Min.	Max.
Institutional Child Delivery	569,031	0.50	0.50	0	1
Violent Events (six months/25 km buffer)	569,031	0.10	1.05	0	73
Violent Events (six months/50 km buffer)	569,031	0.29	2.23	0	129
Violent Events (six months/100 km buffer)	569,031	1.02	5.97	0	264
Urban	569,031	0.26	0.44	0	1
Household Assets Index	558,475	0.23	0.20	0	1
Education (years)	568,730	3.29	4.05	0	23
Never-Mover	344,405	0.41	0.49	0	1
Birth Year	569,031	2004.50	5.72	1989	2014
Birth Order	569,031	3.77	2.50	1	21
Multiple Birth (1 = yes)	569,031	0.04	0.18	0	1

**Table 6:** Descriptive statistics for urban and rural samples

	Number of Observations (births)	Mean	SD	Min.	Max.
A. Urban					
Institutional child delivery	150,249	0.77	0.42	0	1
Violent events (six months/25 km buffer)	150,249	0.02	0.13	0	4.3
Violent events (six months/50 km buffer)	150,249	0.03	0.22	0	7.9
Violent events (six months/100 km buffer)	150,249	0.07	0.42	0	19.8
Household assets index	146,859	0.36	0.24	0	1
Education years	150,112	5.37	4.74	0	23
Never-mover	84,489	0.34	0.47	0	1
Birth year	150,249	2004.14	6.02	1989	2014
Birth order	150,249	3.26	2.27	1	18
Multiple birth (1 = yes)	150,249	0.04	0.19	0	1
B. Rural					
Institutional child delivery	418,782	0.40	0.49	0	1
Violent events (six months/25 km buffer)	418,782	0.01	0.10	0	7.3
Violent events (six months/50 km buffer)	418,782	0.03	0.23	0	12.9
Violent events (six months/100 km buffer)	418,782	0.12	0.66	0	26.4
Household assets index	411,616	0.18	0.17	0	1
Education years	418,618	2.54	3.49	0	20
Never-mover	259,916	0.44	0.50	0	1
Birth year	418,782	2004.03	5.60	1989	2014
Birth order	418,782	3.95	2.55	1	21
Multiple birth (1 = yes)	418,782	0.03	0.18	0	1

## Footnotes

1 See www.measuredhs.com.

2 A potential challenge for our study is that surveys can sometimes be delayed or exclude from the sampling process units in areas that are difficult/unsafe to access because of armed conflict (United Nations 2005). If indeed the most conflict-prone areas in our study are systematically underrepresented by DHS, we assume that this should only underestimate our findings. More importantly, this problem should be less severe given our design because we are using recall data on pregnancies and births covering the five-year period preceding each survey.

3 To ensure respondent confidentiality, each cluster aggregates the average georeferenced location of households. Furthermore, DHS randomly displaces the GPS latitude/longitude positions for all surveys. However, the displacement is relatively small (0–5 km of error) (see http://dhsprogram.com/What-We-Do/GPS-Data-Collection.cfm).

4 See Table 4 in the appendix for a complete list of the countries and surveys included in the analyses.

5 Ideally, we would have preferred to analyze the complete history of all pregnancies/births, but information on institutional births is available for only the five year-period preceding each survey. Furthermore, because the conflict data start in 1989, we censor births prior to this year from the analysis.

6 As an alternative operationalization to institutional births, we also show tests for “birth assistance by medical professional” in Online Resource 1 (see Table S1).

7 A common method to estimate maternal mortality is to use indirect and direct sisterhood methods—that is, to use the information on (maternal) deaths of survey respondents’ sisters. However, this approach requires a very large sample and is not suitable for measuring maternal mortality in small geographical areas (Ahmed and Hill 2010).

8 The unit of analysis is the country-year. The MMR data stem from the World Development Indicators (variable SH.STA.MMRT.NE), and the care variables are based on our own calculations based on the DHS surveys and refer to the percentage of each birth associated with the various services.

9 The map is based on births in 1989–2013 (covering all four Nigerian surveys listed in Table 4 in the appendix). Corresponding maps based on only clusters for each single survey from Nigeria display the same pattern (not shown here).

10 Despite certain data challenges that the UCDP is fully transparent about (see Croicu and Sundberg 2016), we judge the UCDP GED to be of the highest quality available and, for our purposes, better suited than other subnational data, such as the Armed Conflict Location & Event Data Project. For a systematic comparison of the two sources, see Eck (2012).

11 A dyad consists of two conflicting primary parties, or one party killing civilians. In state-based conflicts, at least one party must be the government of a state, but state-based conflicts can involve several dyads if multiple groups oppose the government. Nonstate-based and one-sided violence events are always limited to one dyad (Croicu and Sundberg 2016:15).

12 Hence, if a conflict dyad crossed the 25-deaths threshold in one given year and fewer deaths in either previous or subsequent years, all events belonging to the dyad are included (Croicu and Sundberg 2016:15).

13 All operationalizations are scripted in PostGIS and R for replication purposes.

14 We also show robustness within exogenous fertility windows of three and nine months.

15 Considering our whole estimation sample, 29,076 mothers gave birth both before and after conflict. Most of these mothers had only two children during the period (72 %). We are forced to drop approximately 31,000 children who had no siblings during the period; in total, 62,231 children are included in the sample (see Table S13, Online Resource 1). Roughly speaking, these observations constitute the bulk of the variation used for our estimates (albeit not exactly given that the other observations are used to remove trends, for example). Regressions using only this smaller sample hence yield almost identical albeit somewhat stronger results (results available upon request).

16 The basis for these calculations is as follows. In 2015, the estimated average number of births in SSA was 34,156,809 (United Nations 2015). During the same period, the estimated average population in the region per year was 912,482,643 (United Nations 2015). A rough estimate is therefore that each person corresponds to 0.037 births per year during the period. Using local population estimates from the gridded population of the world (CIESIN 2016), we calculate that an average of 143.6 million people lived within the 50 km buffer zones of a conflict event. The total calculated number of births per year within the areas is therefore 5,312,472 (0.037 × 143,600,000). For ease of calculation, we take the dummy variables in Table S5 (Models 4 and 8) as the basis for the calculation and estimate that 47,812 (0.009 × 5,312,472) fewer births were delivered in a health facility each year as an effect of conflicts. Similarly, an estimated 58,437 fewer births were assisted by medical professionals.
