# Supplementary material for: Organized Violence and Institutional Child Delivery: Micro-Level Evidence From Sub-Saharan Africa, 1989–2014
Source: Demography. 2018 Jun 13;55(4):1295–316. doi: 10.1007/s13524-018-0685-4 (PMC6060989; doi:10.1007/s13524-018-0685-4)
Supplement: Supplementary file 1 — (DOCX 740 kb) [file 13524_2018_685_MOESM1_ESM.docx]

Online Resource 1

Organized violence and institutional child delivery

*Micro-level evidence from sub-Saharan Africa, 1989­–2014*

1. **Robustness tests**

Table S1 Organized violence and child delivery: Different time windows and IVs

|  | (1) | (2) | (3) | (4) | (5) | (6) |
| --- | --- | --- | --- | --- | --- | --- |
| VARIABLES | Institutional child delivery | Institutional child delivery | Institutional child delivery | Birth assistance by medical professional | Birth assistance by medical professional | Birth assistance by medical professional |
|  |  |  |  |  |  |  |
| ln_count3m_50^£^ | -0.010*** |  |  | 0.009*** |  |  |
|  | (0.003) |  |  | (0.003) |  |  |
| ln_count6m_50^££^ |  | -0.009*** |  |  | 0.008*** |  |
|  |  | (0.003) |  |  | (0.003) |  |
| ln_count9m_50^£££^ |  |  | 0.007*** |  |  | 0.005** |
|  |  |  | (0.002) |  |  | (0.002) |
|  |  |  |  |  |  |  |
| Observations | 569,031 | 569,031 | 569,031 | 569,201 | 569,201 | 569,201 |
| R-squared | 0.008 | 0.008 | 0.008 | 0.007 | 0.007 | 0.007 |
| Number of mothers | 390,484 | 390,484 | 390,484 | 390,574 | 390,574 | 390,574 |
| Mother FE | Yes | Yes | Yes | Yes | Yes | Yes |
| Year FE | Yes | Yes | Yes | Yes | Yes | Yes |
| Mean in sample | 0.501 | 0.501 | 0.501 | 0.509 | 0.509 | 0.509 |

Linear regression results. Robust standard errors clustered on DHS primary sampling unit in parentheses. *** p<0.01, ** p<0.05, * p<0.1. ^£^logged number of conflict events within 50 km radius **3** months prior to birth. ^££^logged number of violent events within 50 km radius **6** months prior to birth. ^£££^ logged number of violent events within 50 km radius **9** months prior to birth. Controls for birth order and multiple births not shown.

Table S2 Organized violence and institutional child delivery: Different time windows and radii

|  | (1) | (2) | (3) | (4) | (5) | (6) |
| --- | --- | --- | --- | --- | --- | --- |
| VARIABLES |  |  |  |  |  |  |
|  |  |  |  |  |  |  |
| ln_count3m_25^£^ | -0.009* |  |  |  |  |  |
|  | (0.005) |  |  |  |  |  |
| ln_count3m_100^£££^ |  | 0.006*** |  |  |  |  |
|  |  | (0.002) |  |  |  |  |
| ln_count6m_25^£^ |  |  | -0.008** |  |  |  |
|  |  |  | (0.004) |  |  |  |
| ln_count6m_100^£££^ |  |  |  | 0.007*** |  |  |
|  |  |  |  | (0.002) |  |  |
| ln_count9m_25^£^ |  |  |  |  | -0.005 |  |
|  |  |  |  |  | (0.003) |  |
| ln_count9m_100^£££^ |  |  |  |  |  | -0.005*** |
|  |  |  |  |  |  | (0.002) |
| Observations | 569,031 | 569,031 | 569,031 | 569,031 | 569,031 | 569,031 |
| R-squared | 0.008 | 0.008 | 0.008 | 0.008 | 0.008 | 0.008 |
| Number of mothers | 390,484 | 390,484 | 390,484 | 390,484 | 390,484 | 390,484 |
| Mother FE | Yes | Yes | Yes | Yes | Yes | Yes |
| Year FE | Yes | Yes | Yes | Yes | Yes | Yes |
| Mean in sample | 0.501 | 0. 501 | 0. 501 | 0. 501 | 0. 501 | 0. 501 |

Linear regression results. Robust standard errors clustered on DHS primary sampling unit in parentheses. *** p<0.01, ** p<0.05, * p<0.1. ^£^logged number of violent events within 50 km radius **3** months prior to birth. ^££^logged number of violent events within 50 km radius **6** months prior to birth. ^£££^ logged number of violent events within 50 km radius **9** months prior to birth. Controls for birth order and multiple births not shown.

Table S3 Organized violence and birth assistance by medical professional (BAMP): Different time windows and radii

|  | (1) | (2) | (3) | (4) | (5) | (6) |
| --- | --- | --- | --- | --- | --- | --- |
| VARIABLES |  |  |  |  |  |  |
|  |  |  |  |  |  |  |
| ln_count3m_25^£^ | -0.009* |  |  |  |  |  |
|  | (0.005) |  |  |  |  |  |
| ln_count3m_100^£££^ |  | -0.006*** |  |  |  |  |
|  |  | (0.002) |  |  |  |  |
| ln_count6m_25^£^ |  |  | -0.007** |  |  |  |
|  |  |  | (0.004) |  |  |  |
| ln_count6m_100^£££^ |  |  |  | -0.006*** |  |  |
|  |  |  |  | (0.002) |  |  |
| ln_count9m_25^£^ |  |  |  |  | -0.004 |  |
|  |  |  |  |  | (0.003) |  |
| ln_count9m_100^£££^ |  |  |  |  |  | -0.005*** |
|  |  |  |  |  |  | (0.002) |
| Observations | 569,201 | 569,201 | 569,201 | 569,201 | 569,201 | 569,201 |
| R-squared | 0.007 | 0.007 | 0.007 | 0.007 | 0.007 | 0.007 |
| Number of mothers | 390,574 | 390,574 | 390,574 | 390,574 | 390,574 | 390,574 |
| Mother FE | Yes | Yes | Yes | Yes | Yes | Yes |
| Year FE | Yes | Yes | Yes | Yes | Yes | Yes |
| Mean in sample | 0.509 | 0.509 | 0.509 | 0.509 | 0.509 | 0.509 |

Linear regression results. Robust standard errors clustered on DHS primary sampling unit in parentheses. *** p<0.01, ** p<0.05, * p<0.1. ^£^logged number of violent events within 50 km radius **3** months prior to birth. ^££^logged number of violent events within 50 km radius **6** months prior to birth. ^£££^ logged number of violent events within 50 km radius **9** months prior to birth. Controls for birth order and multiple births not shown.

Table S4 Organized violence and child delivery within different time windows: number of 10 violent events within 50 km radius

|  | (1) | (2) | (3) | (4) | (5) | (6) |
| --- | --- | --- | --- | --- | --- | --- |
| VARIABLES | Institutional child delivery | Institutional child delivery | Institutional child delivery | Birth assistance by medical professional | Birth assistance by medical professional | Birth assistance by medical professional |
|  |  |  |  |  |  |  |
| count3m_50^£^ | -0.014* |  |  | -0.011 |  |  |
|  | (0.008) |  |  | (0.007) |  |  |
| count6m_50^££^ |  | -0.009** |  |  | -0.007* |  |
|  |  | (0.004) |  |  | (0.004) |  |
| count9m_50^£££^ |  |  | -0.005* |  |  | -0.004 |
|  |  |  | (0.003) |  |  | (0.003) |
|  |  |  |  |  |  |  |
| Observations | 569,031 | 569,031 | 569,031 | 569,201 | 569,201 | 569,201 |
| R-squared | 0.008 | 0.008 | 0.008 | 0.007 | 0.007 | 0.007 |
| Number of mothers | 390,484 | 390,484 | 390,484 | 390,574 | 390,574 | 390,574 |
| Mother FE | Yes | Yes | Yes | Yes | Yes | Yes |
| Year FE | Yes | Yes | Yes | Yes | Yes | Yes |
| Mean in sample | 0.501 | 0.501 | 0.501 | 0.509 | 0.509 | 0.509 |

Linear regression results. Robust standard errors clustered on DHS primary sampling unit in parentheses. *** p<0.01, ** p<0.05, * p<0.1. ^£^Number of violent events within 50 km radius **3** months prior to birth. ^££^Number of violent events within 50 km radius **6** months prior to birth. ^£££^Number of violent events within 50 km radius **9** months prior to birth. Controls for birth order and multiple births not shown.

Table S5 Organized violence and child delivery within 50 km radius, different time windows: 1 or more violent events within 50 km radius (dummy)

|  | (1) | (2) | (3) | (4) | (5) | (6) | (7) | (8) |  |
| --- | --- | --- | --- | --- | --- | --- | --- | --- | --- |
| VARIABLES | Institutional child delivery | Institutional child delivery | Institutional child delivery | Institutional child delivery | Birth assistance by medical prof. | Birth assistance by medical prof. | Birth assistance by medical prof. | Birth assistance by medical prof. |  |
|  |  |  |  |  |  |  |  |  |
| conf3m_50^£^ | -0.013*** |  |  |  | -0.011*** |  |  |  |
|  | (0.004) |  |  |  | (0.004) |  |  |  |
| conf6m_50^££^ |  | -0.013*** |  |  |  | -0.011*** |  |  |
|  |  | (0.004) |  |  |  | (0.004) |  |  |
| conf9m_50^£££^ |  |  | -0.011*** |  |  |  | -0.010*** |  |
|  |  |  | (0.003) |  |  |  | (0.003) |  |
| conf12m_50^££££^ |  |  |  | -0.009** |  |  |  | -0.011*** |
|  |  |  |  | (0.003) |  |  |  | (0.004) |
|  |  |  |  |  |  |  |  |  |
| Observations | 569,031 | 569,031 | 569,031 | 569,031 | 569,201 | 569,201 | 569,201 | 569,201 |
| R-squared | 0.008 | 0.008 | 0.008 | 0.008 | 0.007 | 0.007 | 0.007 | 0.007 |
| Number of mothers | 390,484 | 390,484 | 390,484 | 390,484 | 390,574 | 390,574 | 390,574 | 390,574 |
| Mother FE | Yes | Yes | Yes | Yes | Yes | Yes | Yes | Yes |
| Year FE | Yes | Yes | Yes | Yes | Yes | Yes | Yes | Yes |
| Mean in sample | 0.501 | 0.501 | 0.501 | 0.501 | 0.509 | 0.509 | 0.509 | 0.501 |

Linear regression results. Robust standard errors clustered on DHS primary sampling unit in parentheses. *** p<0.01, ** p<0.05, * p<0.1. ^£^1 or more violent events within 50 km radius (dummy) **3** months prior to birth. ^££^1 or more violent events within 50 km radius (dummy) **6** months prior to birth. ^£££^1 or more violent events within 50 km radius (dummy) **9** months prior to birth. ^££££^1 or more violent events within 50 km radius (dummy) **12** months prior to birth. Controls for birth order and multiple births not shown.

Table S6 Baseline result for main variables, number of logged battle deaths within 50 km radius, different months prior to birth

|  | (1) | (2) | (3) | (4) | (5) | (6) |
| --- | --- | --- | --- | --- | --- | --- |
| VARIABLES | Institutional child delivery | Institutional child delivery | Institutional child delivery | Birth assistance by medical professional | Birth assistance by medical professional | Birth assistance by medical professional |
|  |  |  |  |  |  |  |
| bdln3m_50^£^ | -0.003** |  |  | -0.003** |  |  |
|  | (0.001) |  |  | (0.001) |  |  |
| bdln6m_50^££^ |  | -0.004*** |  |  | -0.003*** |  |
|  |  | (0.001) |  |  | (0.001) |  |
| bdln9m_50^£££^ |  |  | -0.003** |  |  | -0.002* |
|  |  |  | (0.001) |  |  | (0.001) |
|  |  |  |  |  |  |  |
| Observations | 569,031 | 569,031 | 569,031 | 569,201 | 569,201 | 569,201 |
| R-squared | 0.008 | 0.008 | 0.008 | 0.007 | 0.007 | 0.007 |
| Number of mothers | 390,484 | 390,484 | 390,484 | 390,574 | 390,574 | 390,574 |
| Mother FE | Yes | Yes | Yes | Yes | Yes | Yes |
| Year FE | Yes | Yes | Yes | Yes | Yes | Yes |
| Mean in sample | 0.501 | 0.501 | 0.501 | 0.509 | 0.509 | 0.509 |

Linear regression results. Robust standard errors clustered on DHS primary sampling unit in parentheses. *** p<0.01, ** p<0.05, * p<0.1. ^£^logged number of battle deaths within 50 km radius (dummy) **3** months prior to birth. ^££^logged number of battle deaths within 50 km radius (dummy) **6** months prior to birth. ^£££^ logged number of battle deaths within 50 km radius (dummy) **9** months prior to birth. Controls for birth order and multiple births not shown.

Table S7 Organized violence and child delivery: Always lived in the cluster or for 5 years

|  | (1) | (2) | (3) | (4) |
| --- | --- | --- | --- | --- |
|  | Institutional child delivery | Birth assistance by medical professional | Institutional child delivery | Birth assistance by medical professional |
| VARIABLES | *never mover* | *never mover* | *5 years* | *5 years* |
|  |  |  |  |  |
| ln_count6m_50^£^ | -0.008 | -0.004 | -0.005 | -0.002 |
|  | (0.005) | (0.005) | (0.004) | (0.004) |
|  |  |  |  |  |
| Observations | 141,629 | 141,639 | 249,555 | 249,616 |
| R-squared | 0.007 | 0.006 | 0.006 | 0.005 |
| Number of mothers | 99,379 | 99,376 | 172,063 | 172,079 |
| Mother FE | Yes | Yes | Yes | Yes |
| Year FE | Yes | Yes | Yes | Yes |
| Mean in sample | 0.417 | 0.418 | 0.421 | 0.423 |

Linear regression results. Robust standard errors clustered on DHS primary sampling unit in parentheses. *** p<0.01, ** p<0.05, * p<0.1. ^£^logged number of violent events within 50 km radius 6 months prior to birth. Controls for birth order and multiple births not shown.

Table S8 Organized violence and institutional child delivery: Extra migration checks

|  | (1) | (2) | (3) | (4) |
| --- | --- | --- | --- | --- |
|  | Institutional child delivery | Institutional child delivery | Institutional child delivery | Institutional child delivery |
| VARIABLES | *question sample* | *difference* | *control* | *5 years* |
|  |  |  |  |  |
| ln_count6m_50^£^ | -0.005 | -0.006 | -0.004 | -0.004 |
|  | (0.003) | (0.003) | (0.004) | (0.004) |
| missing_mig_ln_count6m_50^£^ |  | -0.008 |  |  |
|  |  | (0.005) |  |  |
| never_mover_ln_count6m_50^£^ |  |  | -0.003 |  |
|  |  |  | (0.007) |  |
| less_five_ln_count6m_50^£^ |  |  |  | -0.002 |
|  |  |  |  | (0.008) |
|  |  |  |  |  |
| Observations | 344,405 | 569,031 | 344,405 | 344,405 |
| R-squared | 0.007 | 0.008 | 0.007 | 0.007 |
| Number of mothers | 239,658 | 390,484 | 239,658 | 239,658 |
| Mother FE | Yes | Yes | Yes | Yes |
| Year FE | Yes | Yes | Yes | Yes |
| Mean in sample | 0.457 | 0.501 | 0.457 | 0.457 |

Linear regression results. Robust standard errors clustered on DHS primary sampling unit in parentheses. *** p<0.01, ** p<0.05, * p<0.1. ^£^logged number of violent events within 50 km radius 6 months prior to birth. Controls for birth order and multiple births not shown.

Table S9 Organized violence and child delivery: Clustering at the country level

|  | (1) | (2) | (3) | (4) | (5) | (6) |
| --- | --- | --- | --- | --- | --- | --- |
| VARIABLES | Institutional child delivery | Institutional child delivery | Institutional child delivery | Birth assistance by medical professional | Birth assistance by medical professional | Birth assistance by medical professional |
|  |  |  |  |  |  |  |
| ln_count3m_50^£^ | 0.010*** |  |  | 0.009* |  |  |
|  | (0.003) |  |  | (0.004) |  |  |
| ln_count6m_50^££^ |  | 0.009*** |  |  | 0.008** |  |
|  |  | (0.003) |  |  | (0.003) |  |
| ln_count9m_50^£££^ |  |  | 0.007** |  |  | -0.005 |
|  |  |  | (0.003) |  |  | (0.004) |
|  |  |  |  |  |  |  |
| Observations | 569,031 | 569,031 | 569,031 | 569,201 | 569,201 | 569,201 |
| R-squared | 0.008 | 0.008 | 0.008 | 0.007 | 0.007 | 0.007 |
| Number of mothers | 390,484 | 390,484 | 390,484 | 390,574 | 390,574 | 390,574 |
| Mother FE | Yes | Yes | Yes | Yes | Yes | Yes |
| Year FE | Yes | Yes | Yes | Yes | Yes | Yes |
| Mean in sample | 0.501 | 0.501 | 0.501 | 0.509 | 0.509 | 0.509 |

Linear regression results. Robust standard errors clustered on DHS primary sampling unit in parentheses. *** p<0.01, ** p<0.05, * p<0.1. ^£^logged number of battle deaths within 50 km radius (dummy) **3** months prior to birth. ^££^logged number of battle deaths within 50 km radius (dummy) **6** months prior to birth. ^£££^ logged number of battle deaths within 50 km radius (dummy) **9** months prior to birth. for birth order and multiple births not shown.

Table S10 Organized violence and institutional child delivery: Censoring countries with no organized violence

|  | (1) |
| --- | --- |
| VARIABLES | Only countries with conflict obs |
|  |  |
| Violent events^£^ | -0.0096*** |
|  | (0.003) |
|  |  |
| Observations | 517,858 |
| Number of mothers | 350,876 |
| R-squared | 0.008 |
| Mother FE | Yes |
| Year FE | Yes |
| Mean in sample | 0.478 |

Linear regression results. Robust standard errors clustered on DHS primary sampling unit in parentheses. *** p<0.01, ** p<0.05, * p<0.1. ^£^logged number of violent events within 50 km radius 6 months prior to birth. Controls for birth order and multiple births not shown.

1. **Testing heterogeneity**

Table S11 Organized violence and institutional child delivery, by time periods

|  | (1) | (2) |
| --- | --- | --- |
| VARIABLES | After 2004 | Before 2005 |
|  |  |  |
| Violent events£ | -0.013*** | -0.005 |
|  | (0.004) | (0.005) |
|  |  |  |
| Observations | 309,232 | 259,799 |
| R-squared | 0.009 | 0.008 |
| Number of dfseq | 219,767 | 193,489 |
| Mother FE | Yes | Yes |
| Year FE | Yes | Yes |
| Mean in sample | 0.550 | 0.443 |

Linear regression results. Robust standard errors clustered on DHS primary sampling unit in parentheses. *** p<0.01, ** p<0.05, * p<0.1. ^£^logged number of violent events within 50 km radius 6 months prior to birth. Controls for birth order and multiple births not shown. The two time periods are split based on the median birth year in our sample.

Table S12 Organized violence and institutional child delivery, by service delivery

|  | (1) | (2) |
| --- | --- | --- |
| VARIABLES | High share of deliveries | Low share of deliveries |
|  |  |  |
| Violent events^£^ | -0.013** | -0.002 |
|  | (0.004) | (0.003) |
|  |  |  |
| Observations | 287,339 | 281,692 |
| R-squared | 0.010 | 0.007 |
| Number of dfseq | 201,902 | 195,560 |
| Mother FE | Yes | Yes |
| Year FE | Yes | Yes |
| Mean in sample | 0.655 | 0.345 |

Linear regression results. Robust standard errors clustered on DHS primary sampling unit in parentheses. *** p<0.01, ** p<0.05, * p<0.1. ^£^logged number of violent events within 50 km radius 6 months prior to birth. Controls for birth order and multiple births not shown.

Table S13 Siblings of the same mother who were born before and after violent event(s)

| Number of children per mother | Freq. | Percent |
| --- | --- | --- |
| 2 | 44,651 | 71.75 |
| 3 | 16,011 | 25.73 |
| 4 | 1,408 | 2.26 |
| 5 | 137 | 0.22 |
| 6 | 24 | 0.04 |
| Total | 62,231 | 100 |

1. **Investigating conflict dynamics’ temporal effects on insitutional child deliveries**

We can also test for whether there is a drop in institutional deliveries corresponding to the conflict start more formally by means of a regression discontinuity design. In such analyses identification of causal effects are obtained by assuming a smooth relationship between birth month and institutional deliveries. That is, we allow for the fact that institutional delivery increases over time, but by controlling for this increase on both sides of the conflict date we get an estimate for the discontinuity in the function.

That is, we center the treatment at month zero for the violent event, which yields:

$$Treatment_{i}=\left\{\frac{1\text{ if Months}_{\text{i}}\geq0}{\text{0 if Months}_{\text{i}}<0}\right.$$

The relationship between *Months* and institutional deliveries is assumed to be smooth so that any discontinuity at the threshold can be attributed to the causal effect of the violent event. In our case, the continuous effect of *Months* is controlled for by estimating:

$Maternal\text{ }care_{i}=\alpha+\chi Treatment_{i}+\beta Months_{i}+\lambda Months_{i}*Treatment_{i}+\varepsilon_{i}$.

The smoothness assumption allows us to estimate the difference between two regression functions at month 0. is our parameter of interest. It is identified by separating the continuous function of months from the discontinuity imposed by the treatment (conflict). By including the interaction term between *Months* and *Treatment*, we allow the slope coefficients to differ on each side of the threshold. This is the same as estimating the two regression functions below and calculating the difference in intercepts (*a1-a2*):

$Maternal\text{ }care_{i}=\alpha_{1}+\beta Months_{i}+\varepsilon_{i}$if months $\geq0$, if $\text{Months}_{\text{i}}\geq0$

$Maternal\text{ }care_{i}=\alpha2+\beta Months_{i}+\varepsilon_{i}$if months $\geq0$, if $\text{Months}_{\text{i}}<0$

It is good practice to include a more flexible function of the running variable and we present results with a second degree polynomial. Table S15 present results with a one-degree polynomial and the results are similar. We also add mother fixed effects (and controls for birth year, for birth order, and for being part of a multiple births) to the regressions so that we are only using variation within mothers who gave birth before and after conflict to estimate the effects.

As we have detailed information on the date of birth as well as the date of each violence event, we are also able to explore the timing of the effects. Figure 5 below graphs the share of births that happen in a medical facility at various points in time before and after a given event. Interestingly, there seems to be a jump downwards in institutional deliveries precisely in the month the violent event happens. Then, after approximately three years the share of woman giving birth in a health facility seems to be back at the same level as just before the event. The counterfactual question of what institutional deliveries would have looked like after three years without organized violence is of course difficult to answer but it seems as if the trends before and after are similar around conflicts. Hence, it appears as if the negative effect of organized violence is persistent.

Fig. S1 Maternal health care before and after a violent event


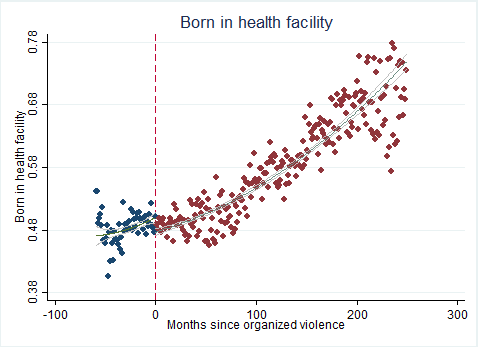


The figure excludes outliers for presentational reasons. A figure with the full sample and for the variable assisted by medical professionals is given in Figure S2.

Fig. S2 Maternal health care before and after conflict


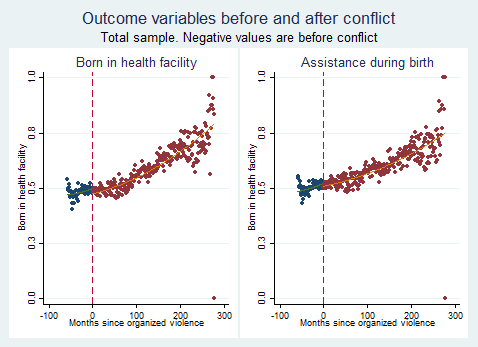


We present the temporal effects more formally through a regression discontinuity design in Table S14.

Table S14 Results with polynomial of degree 1

|  | (1) | (2) | (3) | (4) | (5) |
| --- | --- | --- | --- | --- | --- |
| VARIABLES | 1 year | 2 years | 3 years | 4 years | Full sample |
|  |  |  |  |  |  |
| Treatment | -0.039 | -0.013 | -0.016** | -0.002 | -0.012*** |
|  | (0.026) | (0.013) | (0.008) | (0.006) | (0.004) |
| Months | 0.005* | 0.001 | 0.001** | 0.000*** | 0.001*** |
|  | (0.003) | (0.001) | (0.000) | (0.000) | (0.000) |
| Months* Treatment | -0.003 | -0.000 | -0.000 | -0.001*** | -0.001*** |
|  | (0.004) | (0.001) | (0.000) | (0.000) | (0.000) |
|  |  |  |  |  |  |
| Observations | 47,746 | 88,194 | 124,035 | 153,804 | 266,185 |
| R-squared | 0.024 | 0.014 | 0.010 | 0.011 | 0.009 |
| Number of Mothers | 45,236 | 79,213 | 102,241 | 117,404 | 182,314 |
| Mother FE | Yes | Yes | Yes | Yes | Yes |
| Year Birth order and Multiple FE | Yes | Yes | Yes | Yes | Yes |
| Polynomial degree | One | One | One | One | One |
| Mean in sample | 0.490 | 0.492 | 0.490 | 0.489 | 0.522 |

Robust standard errors in parentheses; *** p<0.01, ** p<0.05, * p<0.1

In the Table S15 below we present results for different time windows. In column one, we restrict the sample to one year before and after conflicts. We see that the jump in the regression function at *Months*=0 is statistically significant, as shown by the coefficient for *Treatment*. Varying the width of the sample window yields similar results as seen in the other columns.

Table S15 Regression discontinuity results with month of birth in relation to conflict

|  | (1) | (2) | (3) | (4) | (5) |
| --- | --- | --- | --- | --- | --- |
| VARIABLES | 1 year | 2 years | 3 years | 4 years | Full sample |
|  |  |  |  |  |  |
| Treatment | -0.086** | -0.039** | -0.024** | -0.012 | -0.012** |
|  | (0.042) | (0.019) | (0.011) | (0.008) | (0.006) |
| Months | 0.016 | 0.005* | 0.002 | 0.001 | 0.001 |
|  | (0.012) | (0.002) | (0.001) | (0.001) | (0.000) |
| Months* Treatment | -0.001 | -0.002 | -0.001 | -0.000 | -0.001 |
|  | (0.015) | (0.004) | (0.002) | (0.001) | (0.000) |
|  |  |  |  |  |  |
| Observations | 47,746 | 88,194 | 124,035 | 153,804 | 266,185 |
| R-squared | 0.025 | 0.014 | 0.010 | 0.011 | 0.009 |
| Number of Mothers | 45,236 | 79,213 | 102,241 | 117,404 | 182,314 |
| Mother FE | Yes | Yes | Yes | Yes | Yes |
| Year Birth order and Multiple FE | Yes | Yes | Yes | Yes | Yes |
| Polynomial degree | Two | Two | Two | Two | Two |
| Mean in sample | 0.490 | 0.492 | 0.490 | 0.489 | 0.522 |

Linear regression results. Robust standard errors clustered on DHS primary sampling unit in parentheses. *** p<0.01, ** p<0.05, * p<0.1.

A natural placebo test to conduct is to pretend that the conflict happened one year before. We see in Table S16 that this produces coefficients that are statistically insignificant, smaller, and that are not even consistent in the sign of the effect. That is, there is no jump one year before when there was no conflict. In total, the results indicate a clear causal effect of conflict on reduced institutional deliveries.

Table S16 Regression discontinuity with a placebo conflict

|  | (1) | (2) | (3) | (4) | (5) |
| --- | --- | --- | --- | --- | --- |
| VARIABLES | 1 year | 2 years | 3 years | 4 years | Full sample |
|  |  |  |  |  |  |
| Placebo (conflict one year before) | 0.003 | 0.000 | 0.002 | -0.008 | -0.007 |
|  | (0.027) | (0.014) | (0.009) | (0.008) | (0.006) |
| Months | -0.002 | 0.001 | 0.000 | 0.000 | 0.001** |
|  | (0.006) | (0.001) | (0.001) | (0.000) | (0.000) |
| Months*Placebo | 0.006 | 0.007** | 0.001 | 0.001 | -0.001** |
|  | (0.013) | (0.003) | (0.002) | (0.001) | (0.000) |
|  |  |  |  |  |  |
| Observations | 46,936 | 87,164 | 120,387 | 144,274 | 266,185 |
| R-squared | 0.031 | 0.017 | 0.013 | 0.013 | 0.009 |
| Number of Mothers | 44,017 | 75,844 | 96,295 | 109,153 | 182,314 |
| Mother FE | Yes | Yes | Yes | Yes | Yes |
| Year Birth order and Multiple FE | Yes | Yes | Yes | Yes | Yes |
| Polynomial degree | Two | Two | Two | Two | Two |
| Mean in sample | 0.494 | 0.490 | 0.487 | 0.487 | 0.522 |

Linear regression results. Robust standard errors clustered on DHS primary sampling unit in parentheses. *** p<0.01, ** p<0.05, * p<0.1.

1. **Included- and non-included events**

In the main paper, Figure 3 visualizes included and non-included conflict events. Events are not included when located outside of the predefined spatial (50 km) and temporal (6 months prior) cutoffs. Also, many locations have numerous DHS surveys, while others have few, decreasing the likelihood of an event to be included. Figure S1 shows the DHS buffers together with the included and non-included events. Color-intensity reveals the frequency of DHS surveys in an area, where darker colors illustrate higher revisit frequency by DHS.

**Fig. S3** Included and non-included events of organized violence, and survey clusters in sample countries, 1989–2014.


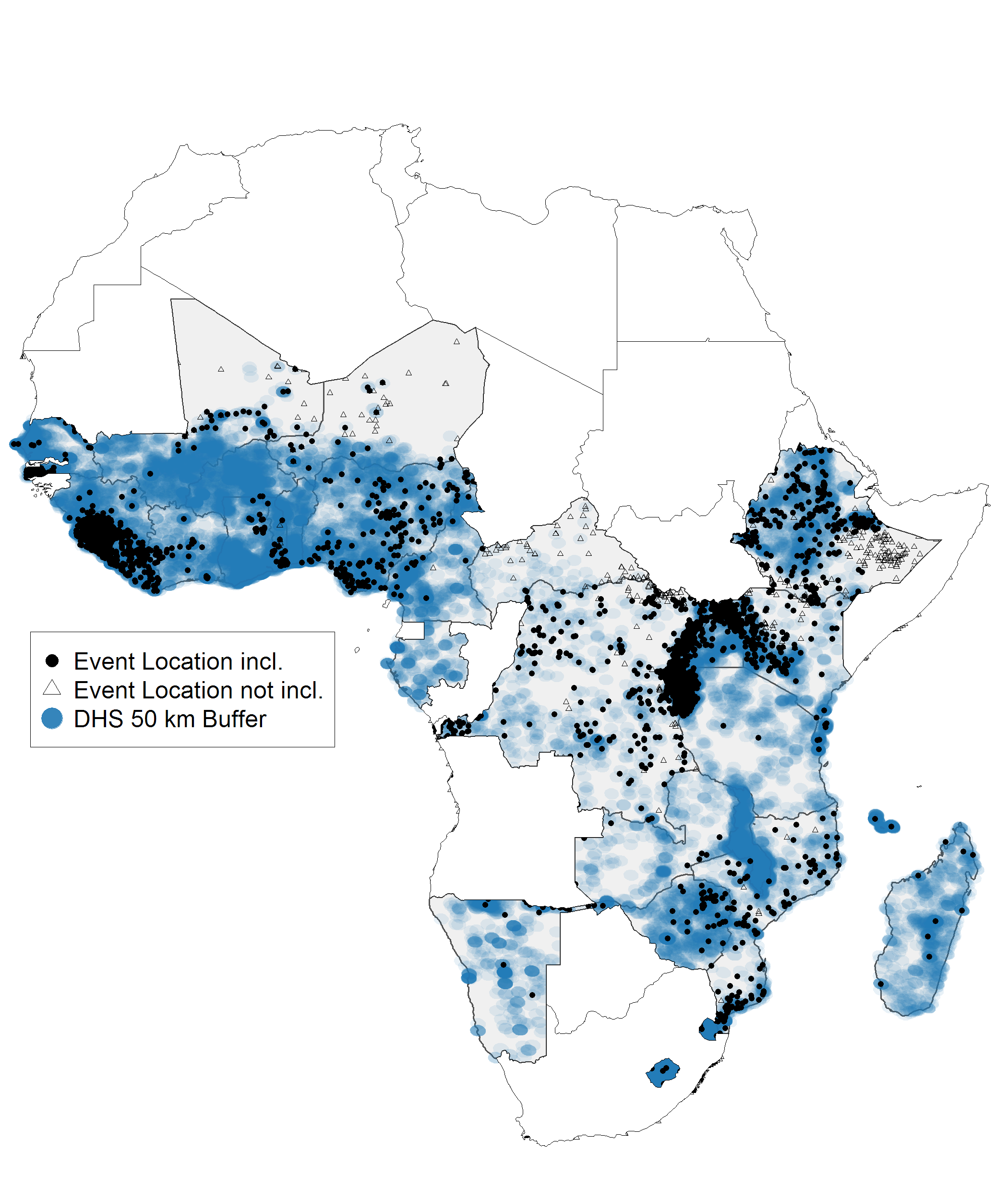


1. **Visualizing conflict heterogeneity**

Figure 3 in the main paper visualizes included versus excluded conflict event locations using the UCDP GED dataset. However, conflict is not homogeneous. UCDP GED separates between three types of conflict; *state-based* conflict, where a government fights a non-state organized actor; *non-state* conflict, where two organized actors engage in violent conflict where neither is the government; and *one-sided* violence, where state or non-state actors attack and victimize civilians. Figure S4 shows the number organized violence events per type per year in the UCDP GED dataset. While the type of conflict varies by year, it also varies by space. Figures S5-S7 shows included and non-included events for the three types of organized violence respectively; state-based, non-state, and one-sided.

Fig. S4 Number of events of organized violence per year, by type


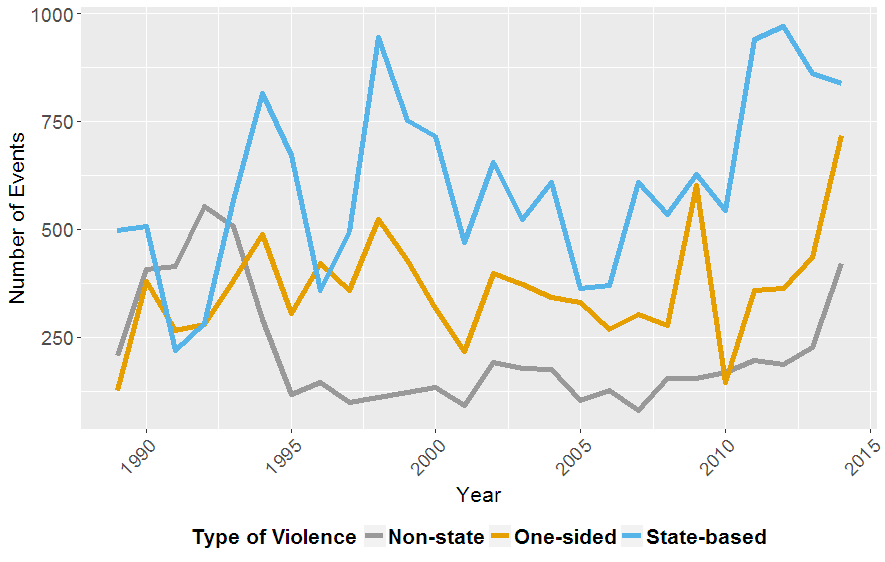


Fig. S5. Included and non-included events, state-based events only


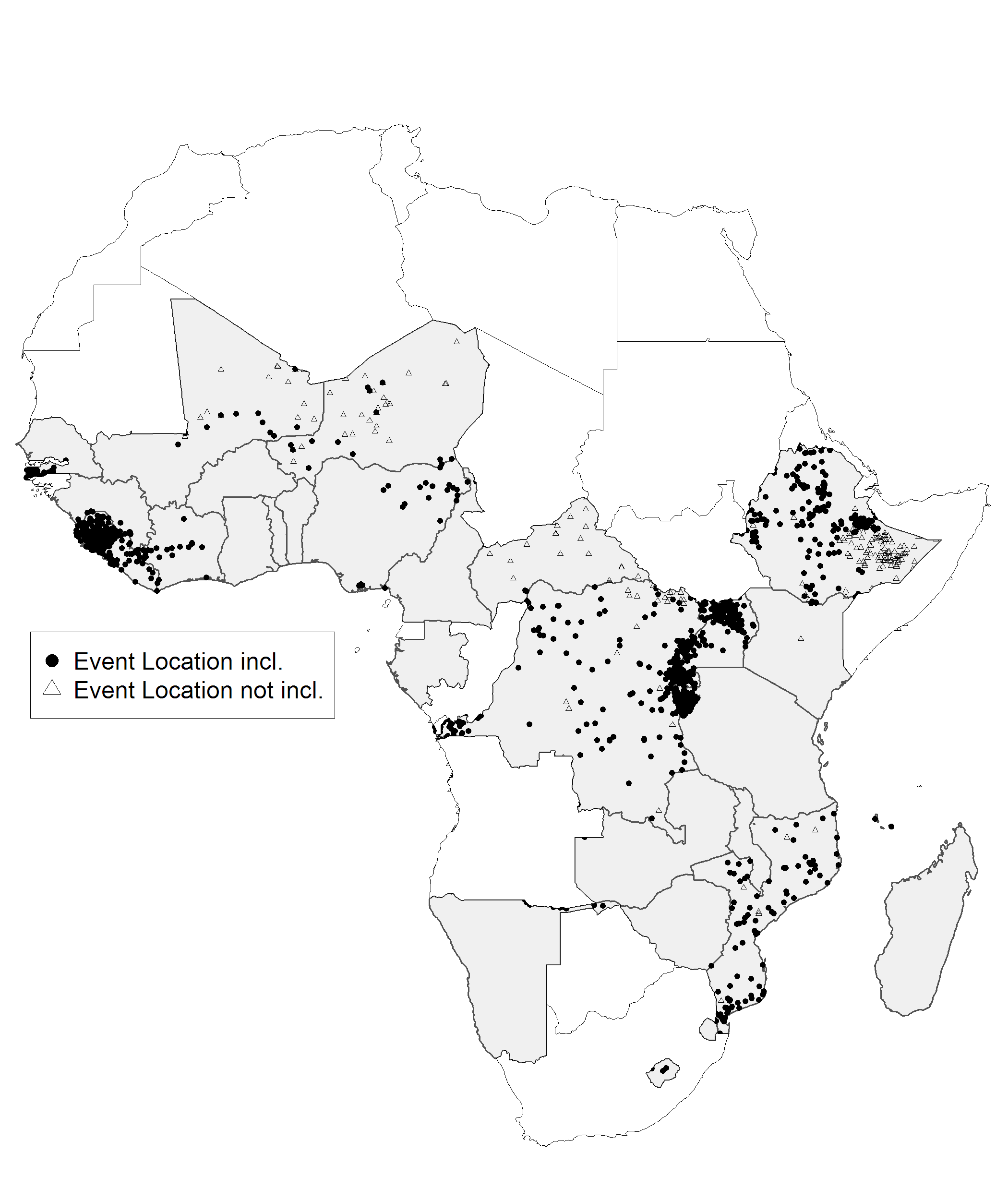


Fig. S6. Included and non-included events, non-state events only


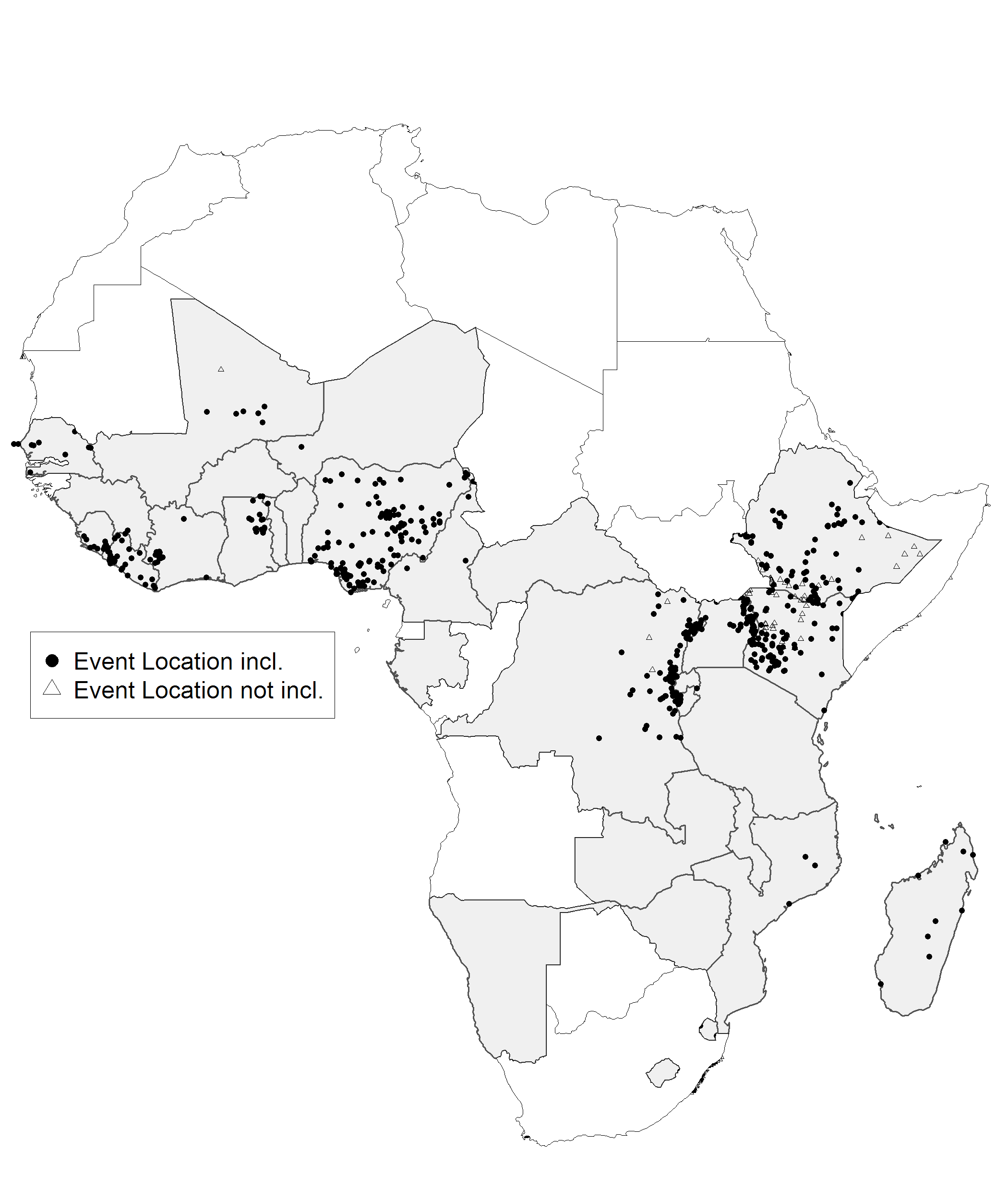


Fig. S7 Included and non-included events, one-sided events only


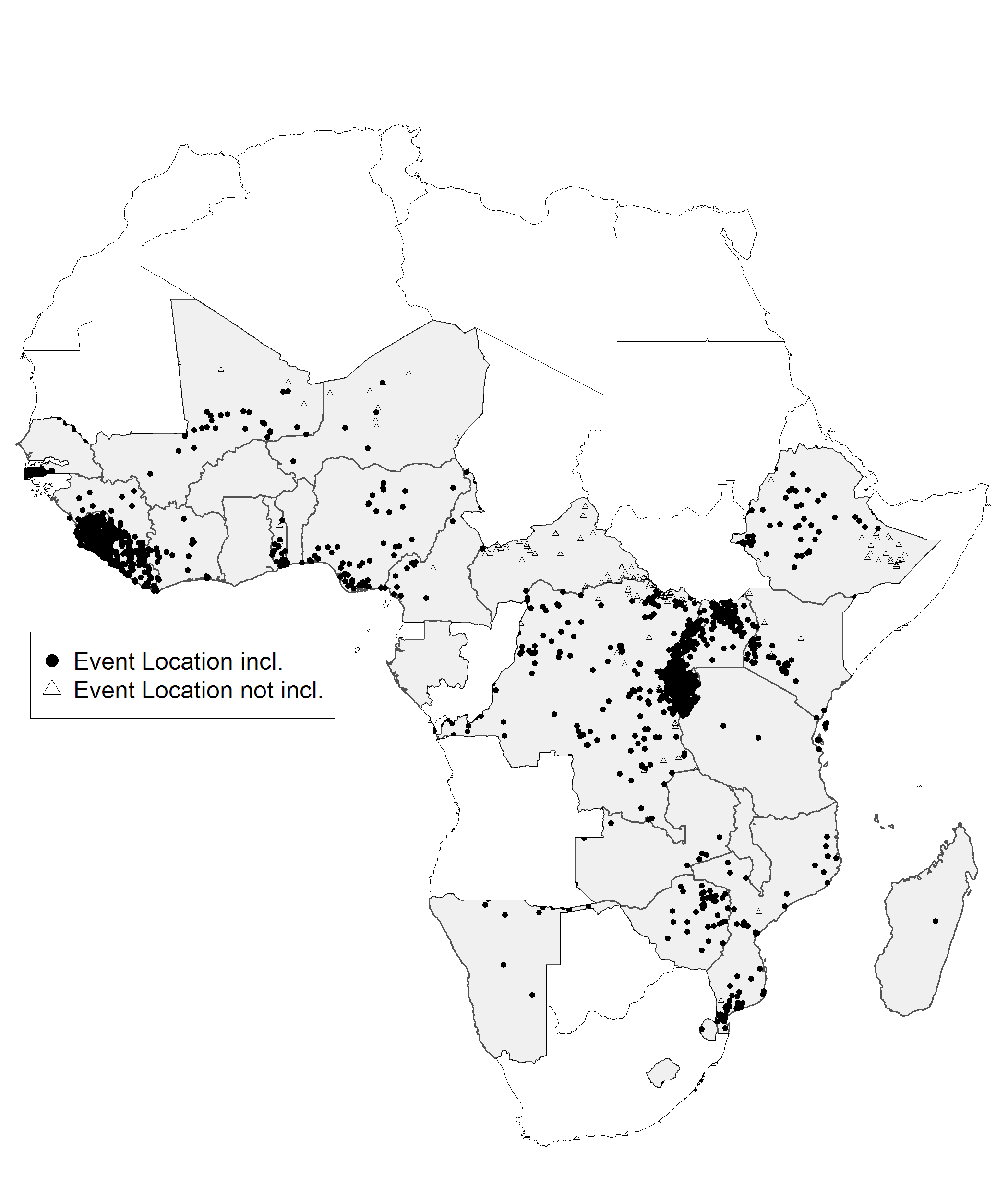


1. **Potential differential effects by conflict type**

Armed conflict is a complex phenomenon and usually consists of several violent events that vary over time and across space, regarding intensity, and in terms of what actors are involved. The UCDP GED dataset distinguishes between three types of organized violence: events between an organized actor and a state government (state-based conflict events), events of violence between two non-state actors (non-state conflict events), and acts of organized violence against civilians, either committed by the state or by a non-state group (Croicu and Sundberg 2016).

*State-based conflict* takes place between two states, or one state and one or more rebel groups. A prominent example is the armed conflict between the Nigerian state and Boko Haram (later the Islamic State in Nigeria). *Non-state conflict* is fought between two organized, armed actors, of which neither is the government of a state. These are typically pastoral conflicts, or regional, ethnic or religious identity conflicts. One example is the conflict between the Kalenjin and Kikuyu ethnic groups in Kenya, which has erupted in electoral violence on several occasions. *One-sided violence* is perpetrated by an organized armed group, either a state’s military forces or a rebel group, against civilians. A well-known case is the Janjaweed militia in Sudan’s attacks on civilians in Darfur from 2003 and onwards.

It is possible that these three forms of organized violence may have different impacts with regard to institutional child delivery. Our expectation is that state-based and one-sided violence should have the strongest effect on institutional births. The former because state-based conflicts typically involve more organized and better equipped conflict parties with greater capabilities to inflict damage on infrastructure, something which may only partly be captured by our intensity measures. In contrast, there may be a weaker effect of non-state organized violence because it often takes place in remote areas where there are fewer hospitals to destroy in the first place. Deliberate attacks on civilians, on the other hand, are expected to have a particularly detrimental effect on institutional births since such attacks generally restrict the movement of civilian populations and also regularly involve targeted sexual violence.

In Table S17 we divide the original independent variable into the three separate sub-types of organized violence. As before, the included events pertain to conflicts for dyads and actors that have crossed the 25 deaths threshold in any year of the UCDP annual data. In line with our expectation, the results are clearly driven by state-based and one-sided violence. For non-state violence, the magnitude of the effect is subtle and not statistically significant. To some extent the difference in results across the various types is likely driven by their prevalence in the data. Most observations did not experience any violence and this is even more so for non-state violence (only 1 percent of the observations experienced a recent non-state event within a 50 km radius). Hence, these disaggregated results should be taken with a grain of salt.

**Table S17** Organized violence and institutional birth: Baseline results[[1]](#footnote-1)

| VARIABLES | (1) | (2) | (3) | (4) |
| --- | --- | --- | --- | --- |
|  |  |  |  |  |
| All violence events | -0.009*** |  |  |  |
|  | (0.003) |  |  |  |
| State-based events |  | -0.012** |  |  |
|  |  | (0.004) |  |  |
| Non-state events |  |  | -0.000 |  |
|  |  |  | (0.005) |  |
| One-sided events |  |  |  | -0.012*** |
|  |  |  |  | (0.003) |
|  |  |  |  |  |
| Observations | 569,031 | 569,031 | 569,031 | 569,031 |
| R-squared | 0.008 | 0.008 | 0.008 | 0.008 |
| Number of mothers | 390,484 | 390,484 | 390,484 | 390,484 |
| Mother FE | Yes | Yes | Yes | Yes |
| Year FE | Yes | Yes | Yes | Yes |
| Mean in sample | 0.501 | 0.501 | 0.501 | 0.501 |

Linear regression results. Robust standard errors clustered on DHS primary sampling unit in parentheses. *** p<0.001, ** p<0.01, * p<0.05, † p<0.1. ^£^ logged number of conflict events within 50 km radius 6 months prior to birth. Controls for birth order and multiple births not shown.

**References:**

Croicu, M. & Sundberg, R. (2016). UCDP GED Codebook version 5.0. Department of Peace and Conflict Research, Uppsala University.

1. Considering our whole sample, 29,076 mothers gave birth both before and after conflict. Most of these mothers had only two children during the period (72 %). In total, 62,231 children were born to those mothers (see Table S13). Roughly speaking, these observations are what constitutes the bulk of the variation used for our estimates (albeit not exactly as the other observations are used to remove trends etc.). Regressions using this smaller sample hence yield almost identical results albeit somewhat stronger (results available upon request). [↑](#footnote-ref-1)
